# Cannabis-Induced Wellens Syndrome: A Rising Cardiovascular Risk

**DOI:** 10.7759/cureus.88068

**Published:** 2025-07-16

**Authors:** Selin Unal, Keston Rattan, Mir Sulayman Khan, Roy Wang, Omar Amro, Inna Bukharovich

**Affiliations:** 1 Internal Medicine, State University of New York Downstate Health Sciences University, Brooklyn, USA; 2 Cardiology, Kings County Hospital Center, Brooklyn, USA

**Keywords:** cad: coronary artery disease, cannabis use, critical care cardiology, ecg interpretation, wellens syndrome

## Abstract

Wellens syndrome is a rare variant of acute coronary syndrome (ACS) that often heralds an impending large anterior wall myocardial infarction. We present the case of a young male with no traditional coronary artery disease (CAD) risk factors. Despite only minimally elevated troponin levels, the patient's smoking history, serial electrocardiogram (ECG) changes, and the emerging evidence linking cannabis use to cardiovascular risk contributed to the timely diagnosis of Wellens syndrome. Although cannabis is increasingly accepted for medical and recreational use, growing concerns have been raised about its potential adverse cardiovascular effects. This case highlights the importance of early identification and prompt intervention. In Wellens syndrome, a conservative approach is associated with poor outcomes, making the decision to proceed with an invasive strategy critical. Understanding the association between cannabis use and myocardial infarction may shape future medical management as cannabis consumption continues to rise.

## Introduction

Wellens syndrome is characterized by significant T-wave abnormalities, particularly deeply inverted or biphasic T waves in leads V2-V3, which indicate proximal stenosis of the left anterior descending (LAD) coronary artery [1]. It was first identified in 1982 and has a prevalence of 14%-18% among patients presenting with unstable angina [2]. Typically affecting middle-aged to elderly male patients with traditional cardiovascular risk factors such as hypertension, diabetes mellitus, dyslipidemia, and tobacco use, the identification of Wellens syndrome in younger, otherwise healthy individuals is rare. While patients may initially respond well to medical management, they generally experience poor long-term outcomes with conservative treatment. Early identification and prompt invasive management, specifically involving coronary angiography and percutaneous coronary intervention (PCI), are crucial to preventing extensive myocardial damage [3]. Emerging data increasingly associate cannabis use with acute coronary syndrome (ACS), including myocardial infarction, arrhythmias, and sudden cardiac death, especially in younger patients lacking traditional cardiovascular risk factors [4,5]. The potential relevance and novelty of cannabis use as a trigger for Wellens syndrome are particularly important given the increasing global prevalence of cannabis consumption [6].

## Case presentation

A 30-year-old male with no significant past medical history presented to the emergency department complaining of progressively worsening left-sided chest pain over the past month, associated with dyspnea, diaphoresis, and chest tightness. The patient reported symptom exacerbation approximately six hours before hospital presentation. The pain was described as constant, having increased in intensity compared to previous chest pain episodes, exacerbated by physical activity and unrelieved by rest. He reported a 15-year history of smoking approximately five marijuana-tobacco mixed cigarettes daily (approximately 20% tobacco and 50% cannabis by weight), confirming recent usage hours before admission. The patient denied other illicit drug use; toxicology screening confirmed cannabis metabolites without other illicit substances detected. He had no family history of cardiovascular disease.

**Figure 1:**
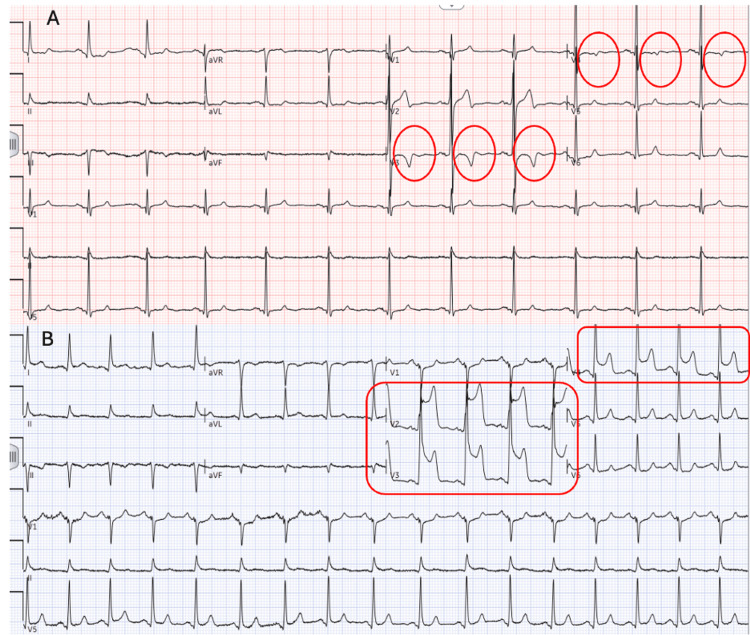
(A) The initial ECG showed biphasic T waves, which suggest myocardial ischemia. (B) A repeat ECG 30 minutes later revealed ST-segment elevation in V2-V4, suggesting acute anterior or antero-septal myocardial infarction in the LAD artery territory. LAD: left anterior descending artery; ECG: electrocardiogram

Upon examination, his initial vital signs were notable for an elevated pressure of 170/140 mmHg and a heart rate of 84 beats per minute. He was afebrile with an oxygen saturation of 100% on room air. Physical examination revealed significant diaphoresis and shortness of breath. However, there were no signs of elevated jugular venous distension or peripheral edema. Cardiac auscultation revealed a normal rate and rhythm, without murmurs, gallops, or rubs. Lungs were clear on auscultation bilaterally. Initial ECG demonstrated sinus rhythm with biphasic T waves in leads V2 and V3, suggestive of myocardial ischemia. A subsequent ECG taken 30 minutes later revealed ST-segment elevation in precordial leads V2 through V4 (Figure 1).

**Figure 2:**
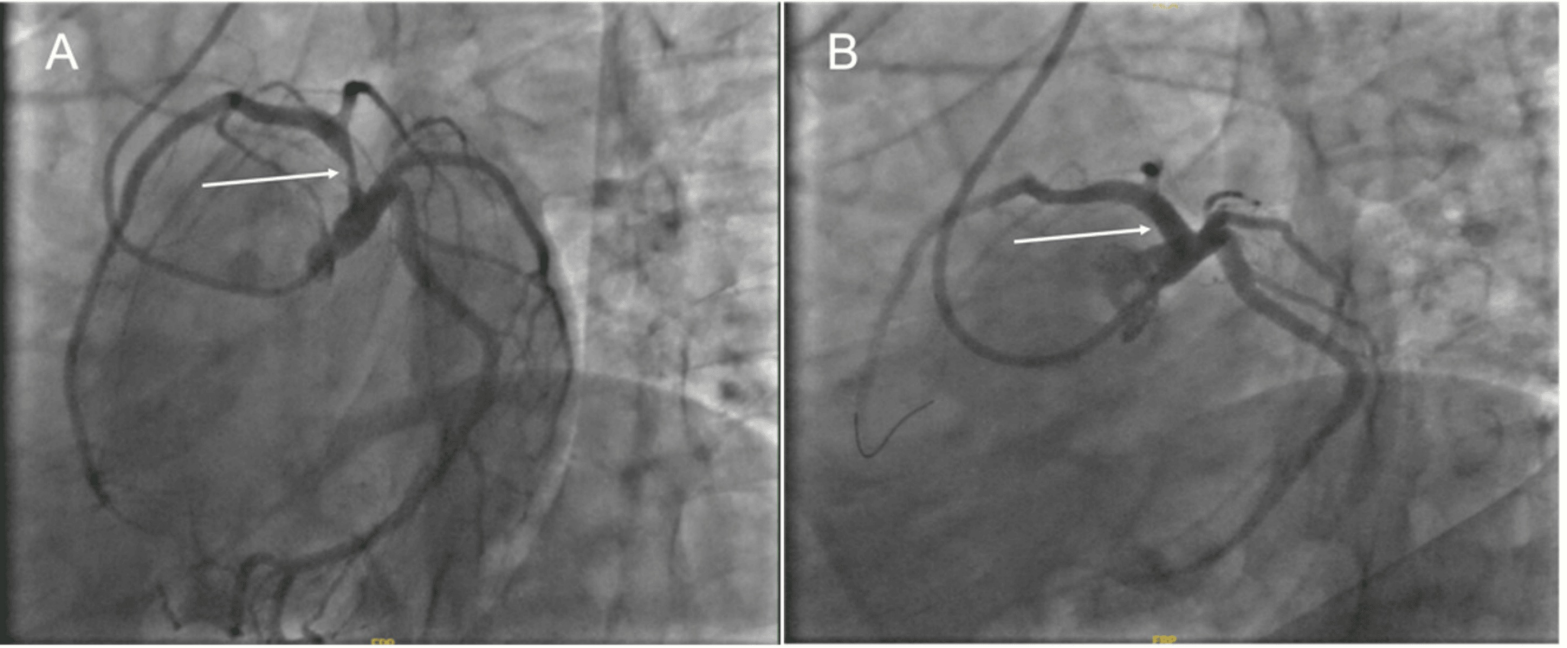
Coronary angiogram. (A) Severe, single-vessel disease involving 80% stenosis of the ostial to proximal LAD. (B) Successful OCT-guided percutaneous coronary intervention (PCI) with placement of a drug-eluting stent. OCT: optical coherence tomography

The laboratory findings were significant for an N-terminal prohormone of B-type natriuretic peptide (NT-proBNP) of 141 pg/mL, high-sensitivity troponin T (hs-cTn) of 30.8 ng/L, and a white blood cell count of 12.61 K/µL (Table 1). The chest X-ray showed clear lungs. The patient was administered aspirin 325 mg and clopidogrel 600 mg and started on a heparin drip. Emergency coronary angiography was performed via the right radial artery using a 5-French Jacky catheter, which revealed an 80% stenosis in the ostial-proximal LAD (Figure 2). Optical coherence tomography (OCT) post-intracoronary nitroglycerin administration revealed an extensive area of lipid-rich plaque with a red thrombus, indicative of an acute thrombotic event superimposed on underlying atherosclerosis.

**Table 1:** Laboratory results on admission

Test	Results	Reference Range	Units
Hemoglobin	17.1	14.0–18.0	g/dL
White blood cell count	12.61	4.50–10.9	K/µL
Platelets	418	130–400	K/µL
Potassium	5.0	3.5–5.0	mmol/L
Magnesium	1.99	1.60–2.60	mg/dL
N-terminal prohormone of brain natriuretic peptide	141	≤125	pg/mL
High-sensitivity troponin T	30.8	0.0–22.0	ng/L

**Figure 3:**
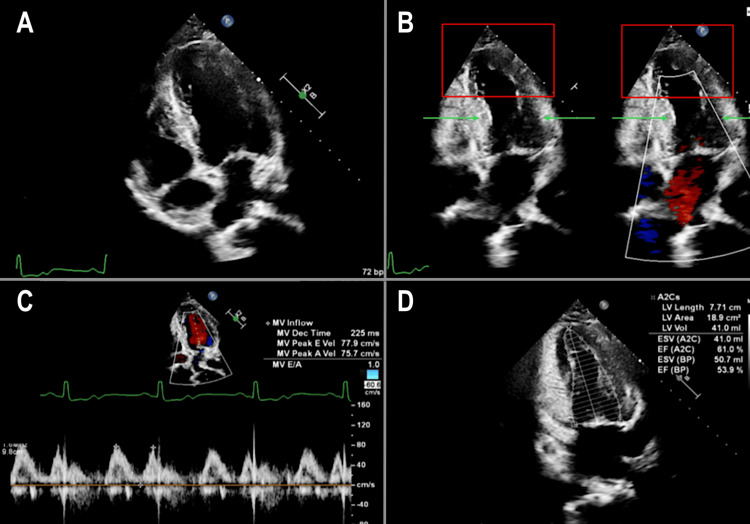
(A) Apical four-chamber view at end diastole. (B) Apical four-chamber view at end systole. Red boxes showed the hypokinesis of the left ventricular apical and apical inferior septal wall. (C) Decreased E to A ratio of the left ventricle. (D) The left ventricular ejection fraction is estimated to be 53% by the Simpson's summation of disks method.

A Xience Skypoint stent (Abbott Vascular, Lake County, IL, USA) measuring 5.0x18 mm was deployed at a maximum pressure of 12 atm, with OCT post-stenting confirming a well-apposed stent and no evidence of edge dissection. The minimal stent area was 14.09 mm², and angiography showed excellent results with 0% residual stenosis and thrombolysis in myocardial infarction (TIMI) 3 flow in the treated vessel. Transthoracic echocardiogram revealed mildly hypokinetic apical septal and severely hypokinetic apical and apical inferior walls without ventricular dilation, minimal left ventricular hypertrophy, normal function in other left ventricular wall segments, and a borderline left ventricular ejection fraction of 53%, findings in keeping with LAD territory infarction. The patient exhibited grade I diastolic dysfunction with impaired relaxation but normal left atrial pressures. The left atrium, right atrium, and right ventricle appeared normal in size and function, with normal valvular structure and movement without evidence of regurgitation or valvular disease (Figure 3).

Upon discharge, the patient was prescribed aspirin 81 mg for three months and ticagrelor 90 mg twice daily for one year, then reduced to 60 mg twice daily indefinitely. He was also discharged on atorvastatin 80 mg daily with the aim of lowering his LDL cholesterol to below 55 mg/dL. His blood pressure resolved during admission, attributing his initial hypertension to pain. He was given cannabis cessation counseling with behavioral support referrals and scheduled for cardiology outpatient follow-up.

## Discussion

Wellens syndrome, also known as LAD coronary artery T-wave syndrome, is a critical pre-infarction condition characterized by distinctive T-wave abnormalities. These changes indicate high-grade stenosis of the proximal LAD artery and carry a significant risk of extensive anterior wall myocardial infarction if not promptly recognized and treated with revascularization [7]. It is typically encountered in middle-aged to elderly male patients with traditional cardiovascular risk factors such as hypertension, diabetes mellitus, dyslipidemia, or tobacco use.

This case is atypical as it describes a young 30-year-old male with minimal traditional cardiovascular risk factors, apart from chronic cannabis-tobacco co-use, who presented with ECG and angiographic findings consistent with Wellens syndrome. This uncommon presentation raises important clinical considerations regarding the potential cardiovascular effects of cannabis, particularly in young adults.

Cannabis and coronary artery disease (CAD): emerging evidence

Cannabis, increasingly legalized in many jurisdictions globally, remains widely used. Global epidemiologic data consistently identify cannabis as a prevalent psychoactive substance [6]. While its psychotropic effects have been widely discussed, growing evidence suggests a significant association between cannabis use and adverse cardiovascular events, including myocardial infarction, arrhythmias, and sudden cardiac death, even in younger individuals without established atherosclerotic disease [4,5].

The primary psychoactive component of cannabis, delta-9-tetrahydrocannabinol (THC), exerts complex cardiovascular effects through its interaction with cannabinoid receptors, primarily cannabinoid receptor 1 (CB1), in the endothelium and myocardium. These interactions have been shown to promote vasoconstriction, oxidative stress, endothelial dysfunction, platelet activation, and a prothrombotic state, mechanisms that may precipitate ACS even in the absence of a significant atherosclerotic burden [5,8].

A landmark case-crossover study by Mittleman et al. demonstrated a 4.8-fold increased risk of myocardial infarction within the first hour following cannabis use [8]. Similarly, Desai et al., using data from the Nationwide Inpatient Sample, found that young adults (18-44 years) with cannabis use disorder had significantly higher odds of acute myocardial infarction compared to non-users, independent of traditional risk factors such as tobacco use [9]. More recent analyses continue to support this association. Ladha et al. reported that recent cannabis use was independently associated with an increased risk of myocardial infarction in adults under 50, reinforcing concerns about its role in premature CAD [10].

A 2025 retrospective cohort study presented at the American College of Cardiology Annual Scientific Session (ACC.25) by Kamel et al. used TriNetX data to evaluate cannabis-related cardiovascular events in adults aged ≤50 years without baseline comorbidities. Among over 4.6 million individuals, cannabis users demonstrated a markedly higher risk of myocardial infarction, ischemic stroke, heart failure, and all-cause mortality compared to non-users, with adjusted odds ratios exceeding six for myocardial infarction alone [11]. These findings underscore the plausibility of cannabis as an independent risk factor for adverse cardiovascular outcomes, even in otherwise healthy populations.

Pathophysiological mechanisms

Several plausible mechanisms have been proposed to explain cannabis-associated ACS and may help contextualize its relationship with Wellens syndrome in our patient: (1) Coronary vasospasm: THC-induced activation of CB1 receptors can trigger coronary vasospasm, particularly in angiographically normal vessels, leading to transient ischemia or supply-demand mismatch [5,12]. (2) Increased myocardial oxygen demand: Acute cannabis use elevates heart rate and blood pressure, increasing myocardial oxygen requirements and potentially unmasking subclinical coronary stenosis [13]. (3) Prothrombotic effects: Cannabis promotes platelet activation and increases circulating procoagulant factors, raising the likelihood of intraluminal thrombus formation, even in non-atherosclerotic vessels [14]. (4) Endothelial dysfunction: Chronic cannabis use impairs nitric oxide bioavailability and promotes oxidative stress, contributing to early endothelial injury and atherogenesis [15].

In our patient, serial ECGs demonstrated dynamic anterior T-wave changes consistent with Wellens syndrome, accompanied by angiographically confirmed 80% stenosis in the ostial-proximal LAD. OCT imaging demonstrated features consistent with atherosclerotic plaque morphology, including a lipid-rich core and red thrombus, confirming the presence of underlying atherosclerosis. The presence of thrombus may have been potentiated by prothrombotic or vasospastic effects of cannabis, but the lesion was definitively atherosclerotic in nature. These findings argue against pure vasospasm and support a multifactorial pathogenesis involving both chronic cannabis-induced endothelial dysfunction with atherogenesis and acute cannabis-induced prothrombotic activity. It is also important to note that while cannabis may have acted as an acute trigger for plaque destabilization or thrombus formation, the chronic co-use of tobacco also likely contributed significantly to atherogenesis in this young patient.

Clinical implications and diagnostic considerations

This case highlights the diagnostic importance of recognizing Wellens syndrome and its characteristic ECG features, particularly in atypical populations. In young patients presenting with chest pain and biphasic or inverted T waves in the anterior leads, early identification is critical, as stress testing may precipitate infarction, and timely revascularization can be life-saving [7].

Furthermore, the case emphasizes the need for a thorough assessment of substance use history in young patients with suspected ACS. Given the increasing potency and widespread availability of cannabis, clinicians should maintain a high index of suspicion for cannabis-associated cardiac events, even in patients who are otherwise healthy. Thorough evaluation of substance use by toxicology screening for objective confirmation should be employed.

While coronary angiography remains the diagnostic gold standard for diagnosing obstructive CAD in suspected Wellens syndrome, intravascular imaging (e.g., OCT or intravascular ultrasound) can clarify lesion etiology, as demonstrated herein. The 2025 guidelines from the American College of Cardiology and American Heart Association explicitly recognize the value of intracoronary imaging, including OCT, for both risk stratification and procedural guidance in patients with ACS, particularly in those with complex or high-risk lesions such as critical proximal LAD stenosis [16]. OCT is highlighted for its unique ability to provide high-resolution visualization of plaque morphology, including the identification of lipid-rich plaques, thin-cap fibroatheroma, and intracoronary thrombus (including red thrombus), all of which are established markers of plaque vulnerability and instability in the context of ACS [17].

Substance cessation counseling with behavioral support referrals should be an integral part of post-myocardial infarction care. Cannabis use, while often underrecognized, may represent a modifiable cardiovascular risk factor that warrants greater attention from both clinicians and public health professionals.

## Conclusions

This case illustrates an uncommon presentation of Wellens syndrome in a young adult, highlighting cannabis use as a significant but contributory cardiovascular risk factor, particularly in the context of concomitant tobacco exposure. Emerging evidence suggests a potential causal role of cannabis in acute coronary events via mechanisms such as coronary vasospasm, increased myocardial oxygen demand, thrombosis, and endothelial dysfunction. Clinicians should remain vigilant in identifying potential cardiovascular risks associated with cannabis consumption, especially in younger individuals who may otherwise appear to have minimal traditional risk factors. Rigorous patient screening, comprehensive lifestyle modification strategies, including targeted cannabis cessation counseling, and aggressive management of co-existing cardiovascular risk factors such as tobacco use are essential components of care. Future prospective observational cohort studies and mechanistic investigations are needed to better delineate the relationship between cannabis use and cardiovascular events, specifically focusing on clarifying causal pathways and the impact of cannabis use on cardiovascular outcomes.
